# Characterizing Sources of Variability in Zebrafish Embryo Screening Protocols

**DOI:** 10.14573/altex.1804162

**Published:** 2018-11-10

**Authors:** Jon T. Hamm, Patricia Ceger, David Allen, Matt Stout, Elizabeth A. Maull, Greg Baker, Amy Zmarowski, Stephanie Padilla, Edward Perkins, Antonio Planchart, Donald Stedman, Tamara Tal, Robert L. Tanguay, David C. Volz, Mitch S. Wilbanks, Nigel J. Walker

**Affiliations:** 1 Integrated Laboratory Systems, Research Triangle Park, North Carolina, USA; 2 Division of the National Toxicology Program, National Institute of Environmental Health Sciences, National Institutes of Health, Research Triangle Park, North Carolina, USA; 3 National Toxicology Program Interagency Center for the Evaluation of Alternative Toxicological Methods, Division of the National Toxicology Program, National Institute of Environmental Health Sciences, National Institutes of Health, Research Triangle Park, North Carolina, USA; 4 Battelle, Life Sciences Research, Columbus, Ohio, USA; 5 Integrated Systems Toxicology Division, National Health and Environmental Effects Research Laboratory, Office of Research and Development, U.S. Environmental Protection Agency, Research Triangle Park, North Carolina, USA; 6 United States Army Engineer Research and Development Center, Vicksburg, Mississippi, USA; 7 Department of Biological Sciences and Center for Human Health and the Environment, North Carolina State University, Raleigh, North Carolina, USA; 8 Pfizer Pharmaceuticals, New London/Norwich, Connecticut, USA; 9 Department of Environmental & Molecular Toxicology, Oregon State University, Corvallis, Oregon, USA; 10 Department of Environmental Sciences, University of California, Riverside, California, USA

## Abstract

There is a need for fast, efficient, and cost-effective hazard identification and characterization of chemical hazards. This need is generating increased interest in the use of zebrafish embryos as both a screening tool and an alternative to mammalian test methods. A Collaborative Workshop on Aquatic Models and 21st Century Toxicology identified the lack of appropriate and consistent testing protocols as a challenge to the broader application of the zebrafish embryo model. The National Toxicology Program established the Systematic Evaluation of the Application of Zebrafish in Toxicology (SEAZIT) initiative to address the lack of consistent testing guidelines and identify sources of variability for zebrafish-based assays. This report summarizes initial SEAZIT information-gathering efforts. Investigators in academic, government, and industry laboratories that routinely use zebrafish embryos for chemical toxicity testing were asked about their husbandry practices and standard protocols. Information was collected about protocol components including zebrafish strains, feed, system water, disease surveillance, embryo exposure conditions, and endpoints. Literature was reviewed to assess issues raised by the investigators. Interviews revealed substantial variability across design parameters, data collected, and analysis procedures. The presence of the chorion and renewal of exposure media (static versus static-renewal) were identified as design parameters that could potentially influence study outcomes and should be investigated further with studies to determine chemical uptake from treatment solution into embryos. The information gathered in this effort provides a basis for future SEAZIT activities to promote more consistent practices among researchers using zebrafish embryos for toxicity evaluation.

## 1 Introduction^1^

Traditional mammalian toxicity tests are time-intensive, expensive, and require both large amounts of test chemical and large numbers of animals (NRC, 2007; Rovida and Hartung, 2009). The cost and time needed to conduct these tests may limit the toxicity data available for the thousands of chemicals in commercial use. The need for toxicity information on these chemicals is driving interest in adopting test methods with higher throughput (Tice et al., 2013; Bugel et al., 2014).

In response to a number of factors, including the National Research Council report *Toxicity Testing in the 21st Century* (NRC, 2007), testing initiatives such as the U.S. government’s interagency Tox21 project (Attene-Ramos et al., 2013; Tice et al., 2013) and the U.S. Environmental Protection Agency’s (EPA) ToxCast^™^ program (Dix et al., 2007) were developed to fundamentally shift current approaches used for hazard identification and characterization. New approaches include the use of high-throughput, cell-based screens to prioritize chemicals for further targeted toxicological testing. However, additional medium- to high-throughput models are needed to provide increased physiological relevance as well as to link *in vitro* observations to molecular, cellular, or physiological effects in whole animals (NRC, 2017).

Zebrafish (*Danio rerio*), which are being developed as physiologically relevant model organisms for medium throughput assays, are a small tropical fish native to the southeastern Himalayan region of Asia and are routinely maintained and bred in a laboratory setting (Lawrence, 2007; Lawrence and Mason, 2012; Dai et al., 2014). Zebrafish have relatively high fertility rates, rapid development with short intergenerational time, and a well-annotated genome with ~70% concordance with mammalian species (Postlethwait et al., 2000; Howe et al., 2013). For these reasons, zebrafish have been used extensively in several fields, including environmental health science (Lieschke and Currie, 2007; Perkins et al., 2013; Aguirre-Martínez et al., 2017; Horie et al., 2017), ecotoxicology (Martins et al., 2007; Almond and Trombetta, 2016; de Oliveira et al., 2016), developmental biology (Solnica-Krezel et al., 1995; Lele and Krone, 1996; Elkouby, 2017) and genetics (Kinth et al., 2013; Varshney et al., 2015; White, 2015; Ceol and Houvras, 2016). Kinth et al. (2013) provides a recent review of literature for use of zebrafish as a model organism, including prominent areas of research, researchers, and research facilities.

Experimental throughput with adult zebrafish is an order of magnitude higher than that of mammals (Collins et al., 2008; Truong et al., 2016). There are, however, ethical concerns with the use of adult zebrafish (Braithwaite and Boulcott, 2007; Sneddon, 2009), and throughput of experiments using adult zebrafish remains below what is needed to screen vast numbers of chemicals. In response to these concerns and in an attempt to reduce, refine, or replace animal use (Russell and Burch, 1992; Tannenbaum and Bennett, 2015), the zebrafish embryo, which is considered to experience little or no pain, suffering, or distress (Strahle et al., 2012) has been investigated as a humane replacement for adult fish (Embry et al., 2010), and adopted for certain acute toxicity testing applications (OECD, 2013b). In the U.S., the National Institute of Health Office of Laboratory Animal Welfare has determined that Public Health Service’s Policy on Humane Care and Use of Laboratory Animals only applies to zebrafish after hatching (Bartlett and Silk, 2016). Similarly, as reviewed in (Sneddon et al., 2017), Directive 2010/63/EU of the European Parliament and of the Council of 22 September 2010 on the protection of animals used for scientific purposes specifies that fish become a protected animal once they are capable of independent feeding, or approximately 120 hours post-fertilization at 28°C. Thus, the use of zebrafish embryos and larvae are in accordance with the 3R principles because it is considered an alternative model in embryonic stages and minimizes the use of mammals (Ducharme et al., 2015).

The ability to generate thousands of developmentally synchronized embryos per day represents a significant advantage of zebrafish over mammals (Westerfield, 2000; Adatto et al., 2011). The small size of zebrafish embryos, approximately 1 to 1.5 mm in diameter (Kimmel et al., 1995; Forbes et al., 2010), makes them easy to maintain and treat in the multi-well plates that are standard for medium-to high-throughput platforms (Yozzo et al., 2013; Leet et al., 2014; Raftery et al., 2014; Vliet et al., 2017). Both the zebrafish embryo and the chorion, the outermost membrane surrounding the zebrafish embryo, are transparent, allowing for direct microscopic observation and evaluation throughout the entire developmental process (Hisaoka, 1958; Hisaoka and Battle, 1958). Additionally, the stages and timing of this process have been thoroughly documented (Kimmel et al., 1995; Westerfield, 2000; Nagel, 2002). The zebrafish embryo shares many characteristics with embryos of other vertebrates. Although zebrafish embryos lack some toxicologically relevant organs such as mammary glands, lungs (Perry et al., 2001), and a prostate gland (Van Slyke et al., 2014), they do possess other toxicologically relevant organ systems such as a liver that expresses cytochrome P450s (Tao and Peng, 2009; Otte et al., 2010; Weigt et al., 2011; Goldstone and Stegeman, 2012) and other metabolic enzymes (Kurogi et al., 2013; Klüver et al., 2014). These characteristics have made the zebrafish embryo an increasingly popular model for developmental toxicology (Dodd et al., 2000; Gunnarsson et al., 2008; Augustine-Rauch et al., 2010, 2016), and tests using zebrafish embryos are included in the battery of assays used in ToxCast (Braunbeck et al., 2005; Padilla et al., 2012a; Truong et al., 2014).

Several different groups have been working to validate zebrafish assays or harmonize zebrafish assay protocols for a number of purposes. Early efforts by the Organisation for Economic Co-operation and Development (OECD) to standardize fish assay protocols included adoption of test guidelines for the fish acute toxicity test (OECD, 1992), the early life stage toxicity test (OECD, 2013a), and the short-term toxicity test on embryo and sac-fry stages (OECD, 1998). These test guidelines allowed for the use of several different fish species including zebrafish. More recently, the fish embryo acute toxicity (FET) test, which assesses acute toxicity in zebrafish embryos up to 96 h post-fertilization (hpf), was approved as OECD Test Guideline 236 (OECD, 2013b; Busquet et al., 2014). One of the groups (Nagel et al., 1991; Schulte, 1994; Nagel, 2002) who worked on developing the zebrafish-specific version of TG 203 (Fish Acute Toxicity Test (OECD, 1992)), known as the 48DarT. This test was further refined by the addition of rat liver microsomes to the test system to allow for the evaluation of proteratogens (Busquet et al., 2008; Weigt et al., 2010). In 2014, a version of the *Dar*T without microsomes was expanded from glass vials into 24-well tissue culture plates and tested in an OECD intra- and inter-laboratory evaluation (Busquet et al., 2014).

In 2010, Brannen et al developed the zebrafish embryo teratogenicity (ZET) assay (Brannen et al., 2010), which used 24-well plates, dechorionated eggs, and embryos exposed statically from 4-6 hpf to up to 5 days post-fertilization (dpf). This model was tested using 34 chemicals with *in vivo* rodent data on developmental toxicity. The model correctly categorized 87% of the chemicals (Brannen et al., 2010) as teratogenic or non-teratogenic. The ZET was further improved in 2010 by the establishment of more standardized morphological scores (Panzica-Kelly et al., 2010).

A consortium of drug development companies developed a basic protocol for the ZET assay; this protocol was then used to test a set of 20 chemicals in four laboratories to evaluate how specific protocol parameters affected assay performance (Gustafson et al., 2012). At the onset of the study, two of the laboratories performed chemical uptake studies, and found that for the chemicals used in the study, the presence or absence of the chorion did not affect chemical uptake. In these two laboratories, the ZET assay protocol was modified to include the chorion (Gustafson et al., 2012). Conversely, one laboratory tested the optimized protocol at the conclusion of Phase I and concluded that dechorionation slightly improved concordance with mouse, rat, and rabbit teratogenicity reference data, but required a complicated assay set-up. Phase II of this effort tested 38 chemicals in two laboratories (Ball et al., 2014). One laboratory used pond-derived fish, while the other used laboratory-bred fish. Both laboratories measured chemical uptake and found that chemicals with low (<5%) uptake were toxic if sufficiently high (1000 μM) concentrations of chemical were applied. Strain differences were not reported as a factor affecting concordance between the two laboratories. In 2015, Panzica-Kelly et al. re-evaluated the optimized protocol used in the Gustafson and Ball studies, using dechorionated embryos as well as embryos with intact chorions and chorions weakened by enzymatic treatment. The embryos with weakened chorions or no chorion exhibited a slight (4%) increase in chemical sensitivity compared to embryos with intact chorions (Panzica-Kelly et al., 2015).

As discussed in Beekhuijzen et al. (2015) and Planchart et al. (2016), the methodology for toxicity tests employing zebrafish embryos varies greatly between laboratories, with differences in the strain of fish used, timing and frequency of exposure, status of the chorion, exposure apparatus, endpoints measured, and scoring of phenotypic alterations. Unfortunately, the pharmaceutical consortium’s studies left many unanswered questions regarding key parameters in the ZET assay protocol. Importantly, the studies did not examine the influence of exposure frequency on toxicity.

The pharmaceutical consortium’s studies also did not resolve important questions surrounding the presence of the chorion. For example, the uptake studies were generated almost exclusively from pharmaceuticals (17/20 chemicals), raising the question of whether the results are valid for other types of chemicals. The fact that the detailed uptake data are not published (Gustafson et al., 2012) makes this question impossible to resolve. Panzica-Kelly et al. (2015) assert that while chorion removal or treatment slightly increased sensitivity, the complexity and set-up time added by this step would be prohibitive for most applications. This review also noted that at least two chemicals identified as teratogens in chorionated embryos were classified as non-teratogens when the chorion was removed or weakened, further complicating an interpretation of the role of the chorion in influencing uptake and toxicity (Panzica-Kelly et al., 2015).

Recognizing both the potential benefits and challenges of using zebrafish and other aquatic models in chemical screening applications, a group of U.S.-based academic and government scientists convened a workshop to identify and propose research initiatives to address the challenges. Among those participating were the laboratories generating data for the ToxCast^™^ screening effort and several additional laboratories using zebrafish as a model organism. The 2014 Collaborative Workshop on Aquatic Models and 21st Century Toxicology was organized by the National Toxicology Program (NTP) Interagency Center for the Evaluation of Alternative Toxicological Methods (NICEATM), North Carolina State University’s Center for Human Health and the Environment, Duke University, EPA, and the U.S. Food and Drug Administration. The purposes of the workshop were to explore and discuss how aquatic models could be used to screen and prioritize chemicals for *in vivo* testing and to assess mechanisms of chemical toxicity to human and environmental health. Discussions focused on how the techniques and knowledge of broad-based, interdisciplinary research could leverage the application of aquatic models to the field of environmental health (Planchart et al., 2016). Workshop participants identified the lack of standardized protocols as an impediment to broader acceptance of these models, and recommended that development of standardized protocols, validation, and subsequent regulatory acceptance would facilitate greater usage.

To address the need for standardized and validated protocols, in 2015 staff from NTP and NICEATM initiated the Systematic Evaluation of the Application of Zebrafish in Toxicology (SEAZIT) to gather the information and data required to achieve a higher degree of standardization for zebrafish embryo protocols. This work would in turn provide a scientific foundation for making decisions on the potential use of zebrafish embryos in chemical safety screening. SEAZIT’s goals include gathering input from zebrafish investigators on protocol elements that are potential sources of outcome variability and facilitating a multi-laboratory evaluation of identified protocol variables. This report summarizes the findings of the initial phase of SEAZIT information-gathering efforts and provides the basis of future activities to explore the potential utility of zebrafish embryo tests in toxicity evaluation.

## 2 Methods and data sources

The initial SEAZIT information gathering efforts focused on scrutinizing current screening methods and determining why investigators using zebrafish embryos in toxicity testing chose their specific experimental protocols. The rationale for this focus was that, given prior experiences described in the Introduction, it seemed appropriate to assess current best practices before proposing new harmonized protocols.

To accomplish this, SEAZIT contacted investigators currently using zebrafish embryos for toxicity testing to assess their willingness to participate in group discussions as an information gathering group (IGG). The eight scientists were selected to include the laboratories generating data in zebrafish embryos for the ToxCast effort and to represent academia, industry, and government. The scientists ultimately selected to participate on the IGG represented six distinct laboratories including two from EPA and two from the U.S. Army (Table 1). SEAZIT team members developed a questionnaire to collect protocol component information from IGG members. The questionnaire is available as supplementary material.^3^ Some of the data collected included zebrafish strains, types of feed, preparation of system water, disease surveillance practices, embryo exposure conditions, and endpoints assessed. The questionnaire responses were transcribed, tabulated, and followed up by individual interviews to clarify details. Five group teleconferences were then held to discuss the group’s findings. SEAZIT team members also reviewed literature from the participating laboratories and citations from PubMed that pertained to issues raised by the IGG.

## 3. Results

### 3.1 Parameters to consider

Despite the growing interest and use of zebrafish in scientific research (Lidster et al., 2017), and the publication of several reviews on general zebrafish care (Lawrence, 2011; Varga, 2011; Lawrence and Mason, 2012), reproductive biology (Lawrence, 2012), and health monitoring (Westerfield, 2000; Collymore et al., 2016), there is still considerable variation in zebrafish husbandry practices in animal facilities and research laboratories. The SEAZIT team discussions with the IGG suggest that husbandry practices and experimental protocol parameters fall into three distinct groups, those which the group felt were unlikely to affect study outcomes and those which we have termed “lesser” and “specific” parameters of concern. The IGG concluded that the lesser parameters of concern are those that theoretically could affect the outcomes of zebrafish experiments, but are not believed to be important sources of inter-laboratory variability. Conversely, variability in the specific parameters of concern could potentially nullify the outcomes and conclusions of embryo experiments. Both lesser and specific parameters of concern are discussed below.

### 3.2 Lesser parameters of concern

#### 3.2.1 Source and strain of fish used

In early zebrafish research, fish were either wild-caught or obtained from commercial breeders or pet shops (Hisaoka, 1958; Hisaoka and Battle, 1958; Hisaoka and Firlit, 1960; Stanton, 1965). In the 1970s and 1980s, efforts to develop specific laboratory strains established the AB strain of zebrafish (Streisinger et al., 1981; Streisinger, 1984; Johnson and Zon, 1999a). Investigators in Germany subsequently developed the Tubingen strain, which has been used extensively for the evaluation of mutations and genetic diversity (Haffter et al., 1996; Geisler et al., 2007; Brown et al., 2012). Many other strains and stocks of zebrafish have since been developed (summarized in Johnson and Zon (1999b)).

It is unclear to what extent observed differences in zebrafish characteristics and behavior are due to genetics, as opposed to individual laboratory and husbandry practices (Ball et al., 2014). Strain-specific behavioral responses to various stimuli have been documented (Vignet et al., 2013; Gao et al., 2016; Quadros et al., 2016; Seguret et al., 2016; van den Bos et al., 2017), including differences in hearing sensitivity (Monroe et al., 2016), visual social preference (Barba-Escobedo and Gould, 2012), and growth performance in response to fasting (Meyer et al., 2013). To date, there is little evidence to indicate the existence of strain differences in sensitivity to toxicants (Chatla et al., 2016), or how genetic differences affect responses to toxicants (Coe et al., 2009). IGG members indicated that in the past, they have used or are using AB, Tubingen, or other strains of zebrafish. However, a common theme in the IGG interviews was that fish used in toxicity studies should be as outbred as possible to avoid the effects caused by line inbreeding, as well as to differentiate zebrafish assays from mammalian-based toxicological screens, which use a very limited gene pool, making zebrafish data more relevant to the human and animal populations which are genetically heterogeneous (Childs and Der Kaloustian, 1968; Brown et al., 2012; Zuberi and Lutz, 2016; Balik-Meisner et al., 2018a,b).

The zebrafish strains used by the IGG are listed in Table 2. Several of the laboratories indicated that in the past they have switched strains to improve fecundity and embryo survival with the 5D strain, a strain originally derived from 5D Tropical (Plant City, FL USA), being a strain they have found to have high fecundity.

#### 3.2.2 Breeding

All the laboratories reported differences in their breeding protocols, driven by individual research needs. The minimum breeding age reported varied both by laboratory and strain. Laboratories generally initiate breeding around four months of age and euthanize breeders at between nine and 24 months of age. Three laboratories mentioned that zebrafish are territorial (Spence and Smith, 2005; Spence, 2011) and that periodically placing previously isolated breeder fish into new groups seems to improve breeding success.

#### 3.2.3. Feed

The adult and larval fish diets reported by laboratories at the time of interview are reported in Table 3. Following group discussion of current practices, several laboratories were investigating changing to the use of Gemma pellet diet (Skettring; Westbrook, ME) without an additional supplementation of live foods to ensure greater definition of the diet. Feed information presented in the rest of this section will reflect the original IGG member reports.

IGG members obtain their fish food from several sources. All IGG members acknowledge that there is a potential for commercial fish foods to be a source of chemical contaminants (Maule et al., 2007; Berntssen et al., 2010; Nácher-Mestre et al., 2014). The majority of commercial suppliers of prepared diets used by IGG members do not provide information regarding pesticide and heavy metal content. One laboratory reported that their supplier tests for both pesticides and heavy metals, while two other laboratories test their diets in-house for mercury, cadmium, and lead content. None of the laboratories had any information about pesticide residue content in feeds.

Five of the six laboratories supplement adult fish diets with brine shrimp (various *Artemia* species ) or other live foods. These laboratories feed rotifers or *Artemia* nauplii to larval fish from hatching until approximately 10 days of age before transitioning to *Artemia* (Seale, 1933; Nash, 1973) and pellet or flake food. IGG members whose laboratories do not supplement adult fish diets with live foods mentioned concerns with the variability in nutrient content and the potential for live foods to be a source of pathogens, heavy metals, and/or pesticide contamination, all of which have been documented by other researchers using aquatic models (Chen and Lru, 1987; Cook et al., 2008; Vikas et al., 2012; Lu et al., 2013; Karlsen et al., 2015). In-house testing of *Artemia* eggs and adults at one IGG laboratory identified mercury present in both. Based on these concerns, that laboratory switched to exclusive use of commercial diets and no longer uses live foods in their fish diet (Miller et al., 2012).

#### 3.2.4 Water

All laboratories use either well water or municipal water in their aquaria. Water is filtered through a reverse osmosis filter and then reconditioned by adding aquarium salt and adjusting the pH before or after it is added to aquaria sump systems. Water circulated within the aquaria systems is mechanically filtered to remove solid particulates, chemically filtered using activated charcoal, and sterilized using ultraviolet light. One laboratory also uses a fluidized sand biofilter prior to charcoal filtration. All laboratories maintain water temperature at approximately 28°C for adult fish and embryos, although two laboratories reduce water temperature to 26°C to slow embryonic development and allow for longer chemical exposures during critical periods of susceptibility. Water temperature is continuously monitored by all laboratories. Monitoring of other parameters such as pH, conductivity, salinity, and ammonia content varied among the laboratories, with some continuously monitoring these parameters and others periodically spot-checking them in an unspecified percentage of experimental tanks using commercial aquarium test kits. All laboratories indicated that they performed daily partial water changes, with some laboratories having automated changes and others performing them manually. Water changes were the most commonly used method to remedy out-of-range water parameters. Water parameters are summarized in Table 4.

#### 3.2.5 Disease and disease monitoring

Five of the six laboratories perform routine disease monitoring; three of these use sentinel fish in this process. Diseases routinely monitored include mycobacteriosis, and microsporidiosis, as well as fresh water velvet disease arising from parasitism by *Piscinoodinium*. Several common fish diseases (Matthews, 2004; Murray et al., 2011; Whipps et al., 2012) have been observed in the IGG facilities (Table 5). Disease diagnosis is performed by trained laboratory staff, and confirmed, if necessary, by a veterinary pathologist. Diseased fish are euthanized and the tanks these fish resided in closely monitored. Several IGG members use quarantine procedures for any newly acquired fish to prevent introduction of disease into breeding stocks, and they strongly recommended this practice be used for all laboratories.

#### 3.2.6 Embryo exposure conditions

Exposure initiation time varies situationally within laboratories as well as across laboratories. The most common time to initiate exposure reported by IGG members is 5–6 hpf with a range from as early as 0.75 hpf to as late as 24 hpf. Decisions about when to initiate and how long to expose embryos to the test chemical are often driven by the endpoint of interest. For example, exposure on or after 24 hpf can increase the chance of finding true vascular disruption rather than vascular disruption secondary to gross morphological malformation (McCollum et al., 2016). One laboratory indicates that beginning exposure at 5–6 hpf allows for selection of embryos that appear to be developing normally, thus reducing background abnormalities. Another laboratory initiates exposure at 24 hpf so that they can identify fish that fail to survive mechanical removal of the chorion.

While the FET Test, which measures acute lethality (OECD, 2013b; Braunbeck et al., 2015) established a defined chemical exposure regime, the period of exposure for other toxicological applications varies. Exposure lengths reported by the IGG range from 4 to 144 hours, depending on the endpoints of interest.

**Fig. 1:**
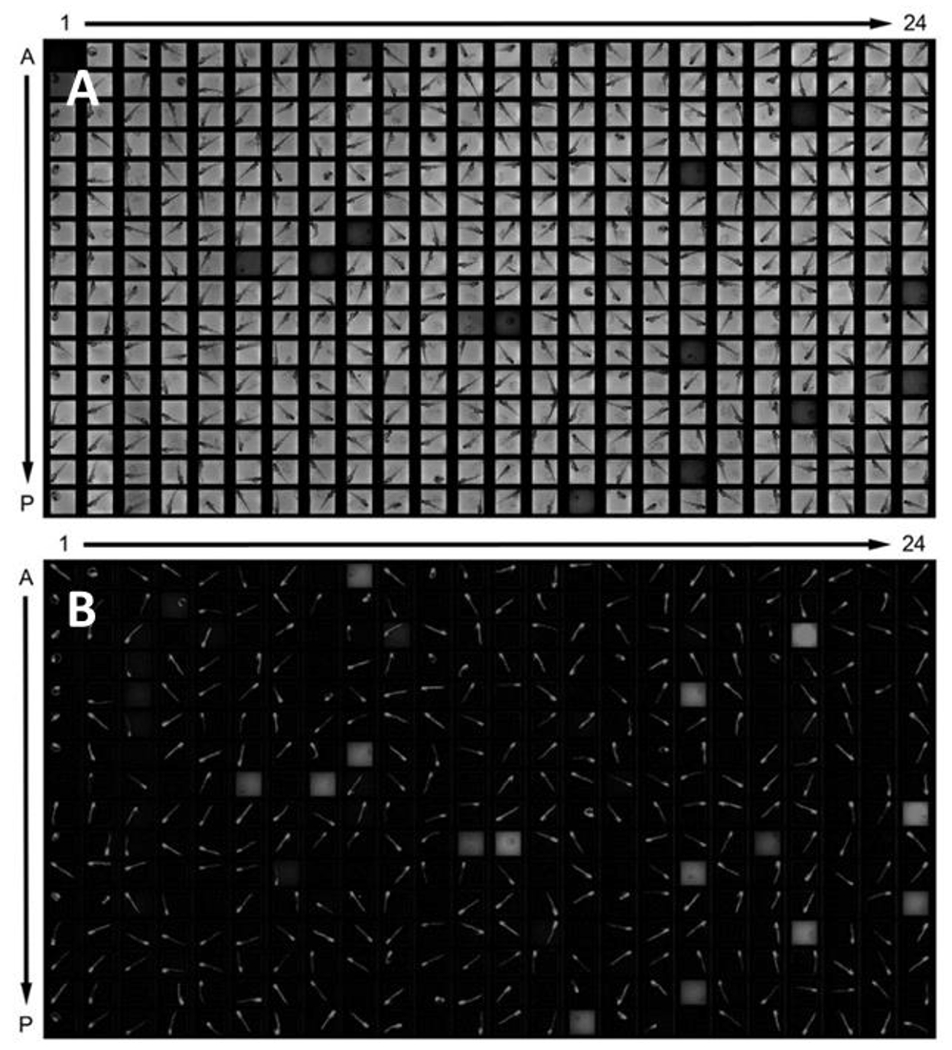
Zebrafish embryos in a 384-well tissue culture plate Photographs of transgenic fluorescent zebrafish embryos taken under a microscope using transmitted light in the top panel (A) and using fluorescence capture in the bottom panel (B). Images are captured simultaneously. One zebrafish embryo is immersed in 50 μL of embryo media in each 3X3 mm well of the 384-well tissue culture plate. The embryos in this image were placed in the well at 5 hpf and the image was taken at 72 hpf.

**Fig. 2:**
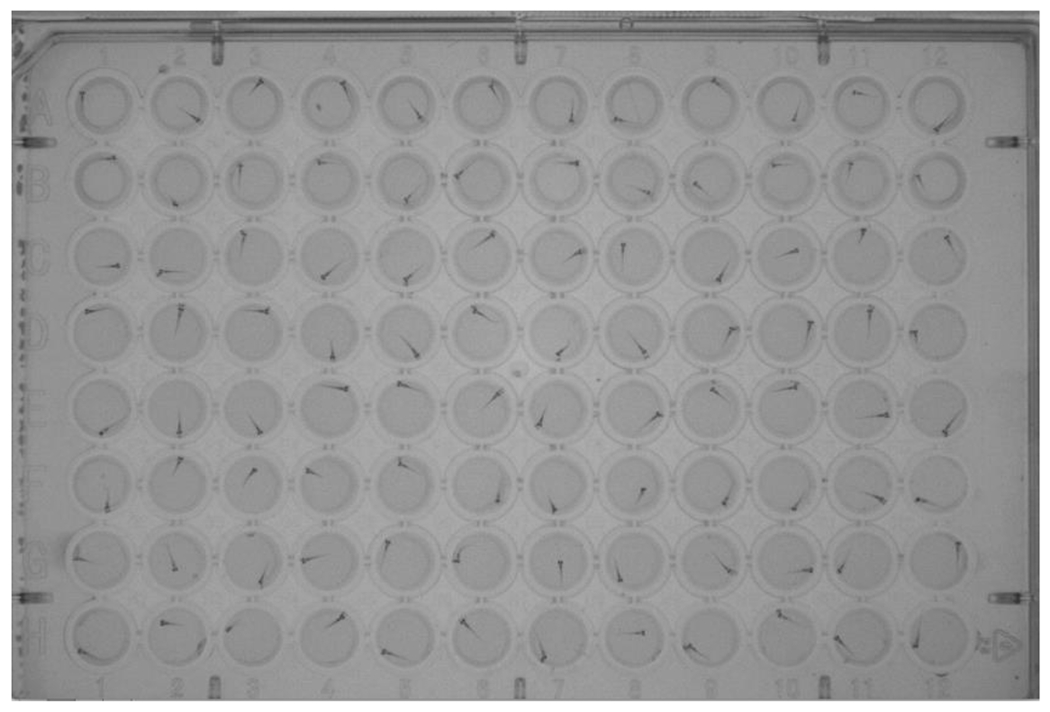
Zebrafish embryos in a 96-well tissue culture plate Photograph of zebrafish embryos in a 96-well tissue culture plate, taken under a microscope using transmitted light.

Embryos are generally placed in multiwell plates for chemical exposure and evaluation. The size of plates used, volume of media per plate, and the number of embryos per well are listed in Table 6. Representative images of zebrafish embryos in 384-well and 96-well tissue culture plates are provided in Figures 1 and 2. Two laboratories place the embryos in the multiwell plates into dark incubators; other laboratories, expressing concerns about circadian rhythms, house their embryos in a controlled light/dark environment, with 14 hours of light and 10 hours of darkness.

The medium used to grow the embryos varies. Three laboratories use E2 medium, with one of these supplementing the medium with methylene blue to inhibit fungal growth. Of the other three laboratories, two use E3 medium (Nüsslein-Volhard and Dahm, 2002), and one uses Hanks’ Balanced Salt Solution.

Solvents used for chemical formulations included dimethyl sulfoxide (DMSO), water, and ethanol. DMSO, the most commonly used solvent, was used in final concentrations ranging from 0.1% to 1%. There are concerns that DMSO may interfere with xenobiotic metabolism (David et al., 2012), alter locomotor activity in zebrafish larvae (Chen et al., 2011), and cause proteotoxic and embryotoxic effects (Hallare et al., 2006). To minimize these potential issues, laboratories prefer to keep DMSO concentrations to less than 1%, and none have observed any behavioral or developmental effects at that level. Two laboratories mentioned concerns that test chemicals may change the pH of the exposure solution, which may alter the bioavailability of the chemical (Erickson et al., 2006; Armitage et al., 2017), or may potentially be fatal to the embryos exposed to pH extremes. The FET test prescribes that “the test solution should be in the range of 6.5 to 8.5 and further states that it “not vary within this range by more than 1.5 units during the course of the test. If the pH is not expected to remain in this range, then pH adjustment should be done prior to initiating the test. The pH adjustment should be made in such a way that the stock solution concentration is not changed to any significant extent and that no chemical reaction or precipitation of the test chemical is caused (OECD, 2013b).” Only one IGG laboratory stated that they measure and adjust the pH of dosing solutions and noted that they have found toxicity can be alleviated once pH was adjusted closer to neutral.

#### 3.2.7 Physio-chemical Properties

The factor universally agreed as impacting exposures was chemical solubility in zebrafish embryo medium. IGG members typically consider a chemicals LogP, the affinity of a chemical for either aqueous and lipophilic solvents (usually octanol and water). This parameter is presumed to affect chemical uptake by adult zebrafish and embryos based on the assumption that highly water-soluble (i.e., hydrophilic) chemicals will stay in the water column and are unlikely to cross the lipid-rich cell membrane (Gustafson et al., 2012; Padilla et al., 2012b; de Koning et al., 2015; Ducharme et al., 2015) Conversely, lipophilic chemicals will more easily transverse cell membranes gaining entrance to the organism. The IGG investigators stated that extremely water-soluble chemicals are likely to produce false negative results due to little to no uptake, while insoluble chemicals cannot be tested in an aqueous exposure. One laboratory visually checks the wells of test plates under a microscope for precipitation from DMSO solutions as an indication of low solubility, and discontinues the experiment if it is observed. Another laboratory continues to conduct a test in the presence of chemical precipitation on the assumption that at least some of the chemical will partition into the culture media and expose the zebrafish embryos; however, at that point it is extremely difficult to determine the exposure concentration. The IGG members agree that logP influences uptake, but does not necessarily determine toxicity; however, they suggest that under repeat-dosing scenarios, logP appears to correlate with potency.

High volatility, a factor raised during the 2014 workshop as a physio-chemical property that could render a chemical unsuitable for testing, was not considered a limiting factor as all laboratories had contingency plans in place, e.g., sealing test plates for use with very volatile chemicals.

#### 3.2.8 Test plate controls and acceptability criteria

All the IGG members indicate that they have experimental acceptance criteria based on fecundity and fertility. These criteria are evaluated before eggs are placed in the multiwell test plates. All laboratories monitor the number of eggs produced and fertilized per spawn. They also record the proportion of viable eggs, i.e., eggs that are transparent, with no sign of coagulation (OECD, 1998). All IGG members state that if any of these parameters are abnormal, the experiment is discarded. However, only one laboratory has a minimum acceptable viability level, which they set at 70%.

All investigators stress the importance of producing as many fertilized, viable, “normal” eggs as possible. Furthermore, they indicate that reduced fecundity and/or egg quality is often the first sign of husbandry troubles in an aquaculture facility. One investigator notes that, in their laboratory’s experience, the first parameter their laboratory staff check when egg production decreases among laboratory fish is the quantity and quality of the diet. Next, they investigate fish stressors such as water quality fluctuations or altered light/dark cycles. Such stressors can also negatively affect egg production and could eventually lead to illness and death.

Among the laboratories surveyed, use of positive controls and the chemicals used varies broadly. Two laboratories found after multiple years of testing that use of positive controls failed to add value to their assays, and thus discontinued using them. One laboratory uses different positive controls based on the chemical class of the study chemicals, i.e., 2,3,7,8-tetrachlorodibenzodioxin for chemicals that interact with the aryl hydrocarbon receptor. One laboratory uses chlorpyrifos at two different concentrations, one selected to be acutely toxic, and the other increasing malformations. One laboratory uses 3,4-dichloroaniline, the recommended control for the FET test (OECD, 2013b). This laboratory reports that the onset of exposure affected the toxicity of 3,4-dichloroaniline, with exposure beginning at 3 hpf causing increased mortality, while exposures beginning at 6 hpf causes increased malformations (Scheil et al., 2009). Finally, one laboratory reports that they routinely use either thiram or dithiocarbamate.

Regarding use of negative control chemicals, only one laboratory indicates they run simultaneous exposure of a negative control chemical with the choice of the negative control dependent on what they were evaluating. Another laboratory uses embryo rearing media as the negative control, while the remaining laboratories utilize a solvent-only exposure as a negative control.

All participating laboratories use in-house test plate acceptability criteria for each study that assess mortality, malformations, or both in solvent control-exposed embryos. The percentage of live embryos required for an acceptable experiment varies between laboratories with the most common cutoff being 85%.

#### 3.2.9 Endpoints and data collection

The endpoints assessed, methods of data collection, and criteria used to evaluate endpoints vary among all laboratories. The most commonly collected endpoints include mortality, edema (pericardial, yolk sac, and other sites), skeletal malformations (including the spine and jaw), and a measure of total length are evaluated to some degree by all laboratories. Spontaneous tail movement at 24 hours and heart beat are assessed by a number of laboratories, as are swim bladder inflation and hatching in laboratories that use chorionated embryos.

The interrelatedness of endpoints is an area of active investigation. One investigator mentioned that, in their laboratory’s experience, swim bladder inflation, edema, and failure to thrive are all signs of generalized toxicity. These findings are sequential: the edema develops first, the swim bladder then fails to inflate, and ultimately the larva fails to thrive. Of note, a meta-analysis by Ducharme et al. (2013) found that altered hatching rate correlated to 20 other endpoints, including several gross morphology endpoints such as curvature of the spine, changes in size, and yolk sac edema, as well as several signaling pathway changes. Recently, Zhang et al. (2017) used a Bayesian method to analyze toxic responses to a large set of chemicals using 17 phenotypic alterations in zebrafish to quantify endpoint utility. Their results suggest that this approach improves identification of significant morphological effects and that a developmental cascade may be evaluated by analyzing the relationships among endpoints.

Approaches to data collection vary. The 2014 workshop featured several presentations of results obtained using automated image capture systems. IGG members report experience with several of these systems, including Array Scan (Cellomics, Inc., Pittsburgh, PA), Noldus System DanioVision with the DanioScope (Noldus Information Technology Inc. Leesburg, VA), VAST System (Union Biometrica, Holliston, MA), Molecular Devices ImageXpress (Molecular Devices, Sunnyvale, CA), and custom-built laboratory imaging systems. All of these systems use proprietary image collection and analysis software. One IGG member stated that their laboratory’s evaluations require that the embryo be assessed from multiple orientations; at the time of these interviews, none of the available imaging systems allowed for this flexibility, so they are continuing to evaluate embryos manually. Other IGG members point out that several endpoints such as swim bladder inflation and head size cannot be measured by some of the automated systems. Some imaging systems require operators to manually orient agarose-embedded embryos before imaging, adding time and cost to the assay, as well as risking damage to the embryo during manipulation. Two participating laboratories use the VAST System, which addresses this issue by automatically loading zebrafish from reservoirs or multiwell plates and positioning and rotating them for imaging without damage to the embryo (Pardo-Martin et al., 2010).

### 3.3 Specific parameters of concern

#### 3.3.1 Static vs. static renewal

The IGG investigators agreed that the method of exposing embryos to the test chemical is a key factor in experimental design. This parameter might reasonably be expected to influence the amount of chemical in the medium and in the embryo, and variability can affect the outcomes and conclusions of exposure experiments.

The three methods used to expose fish to test chemicals are static, static-renewal (i.e. semi-static), and flow-through (OECD, 2002). In the static method, the test chemical is added to the culture medium at the beginning of the experiment, and this exposure solution is used for the duration of the experiment without replacement of the culture medium or replenishment of the test chemical. The static-renewal method designates certain time points during the experiment at which the test solution is replaced with a fresh mixture of culture medium and test chemical; this is most typically done every 24 hours. The flow-through method requires a system in which test solution can be constantly circulated through the exposure environment, with monitoring and adjustment of test chemical concentration, simulating real-world aquatic exposure conditions.

Most zebrafish embryo chemical toxicity testing protocols use either the static or static-renewal exposure methods. As reviewed in Beekhuijzen et al. (2015), the choice of exposure method can be based on the physicochemical properties of the study chemicals or practical considerations of use in the laboratory. Static exposure protocols are easy and economical, as the test chemical does not need to be freshly measured out for each chemical exposure, and there is no additional time or labor required to replace the medium. Static-renewal protocols can help mitigate the loss of chemical due to volatilization, decomposition, or adherence to the exposure vessel while allowing for the use of multiwell test plates. Although Lammer et al. (2009) developed a flow-through setup using 24-well plates as test chambers, flow-through is generally incompatible with the use of tissue culture plates and can require significant amounts of test chemical.

One IGG member reported that in their laboratory’s experience, the static-renewal method increased death and malformation rates in their controls due to manipulation of the embryos. Their (unpublished) evaluation of chemical uptake on a panel of over 100 chemicals found that, with a single exception, all chemicals were detected in the embryos to some extent, indicating that renewing test chemical was not necessary to achieve uptake.

Another investigator, whose laboratory uses 384-well test plates in its exposure protocol, indicated that renewal is not possible in their setup due to an inability to find pipette tips fine enough to fit into the wells and aspirate the media without also inadvertently removing embryos. For that group, the advantages of using a 384-well plate platform (i.e., quadrupling experimental N, using less test chemical, and more efficient high content time-lapsed imaging) outweigh any potential concerns about medium renewal.

Some IGG members reported that they only use the renewal method if significant metabolism of the test chemical by the zebrafish embryos is anticipated. One of the laboratories described a partial renewal protocol in which 40% of the medium in a well was replaced, thereby decreasing the chance of embryo desiccation by removing too much medium or accidentally disturbing the embryo with the pipette tip. This approach is required for dechorionated embryos, although with chorionated embryos, 100% of the media can be replenished using mesh-bottom inserts to lift out the embryos, blot, and return to fresh exposure media.

As a group, four of the IGG members reported that their laboratories exclusively use static exposures, while three of the laboratories use both static and static-renewal exposures. There was a difference of opinion about how the use of static versus static-renewal methods might influence toxicity. Some members stated that repeat exposure is capable of producing exceedingly high body burdens while others noted that false negative results are more likely in a single-static-exposure scenario when uptake is limited. IGG members agreed that a better understanding of toxicokinetics in zebrafish would help clarify the effect of different exposure methods on toxicity.

**Fig. 3:**
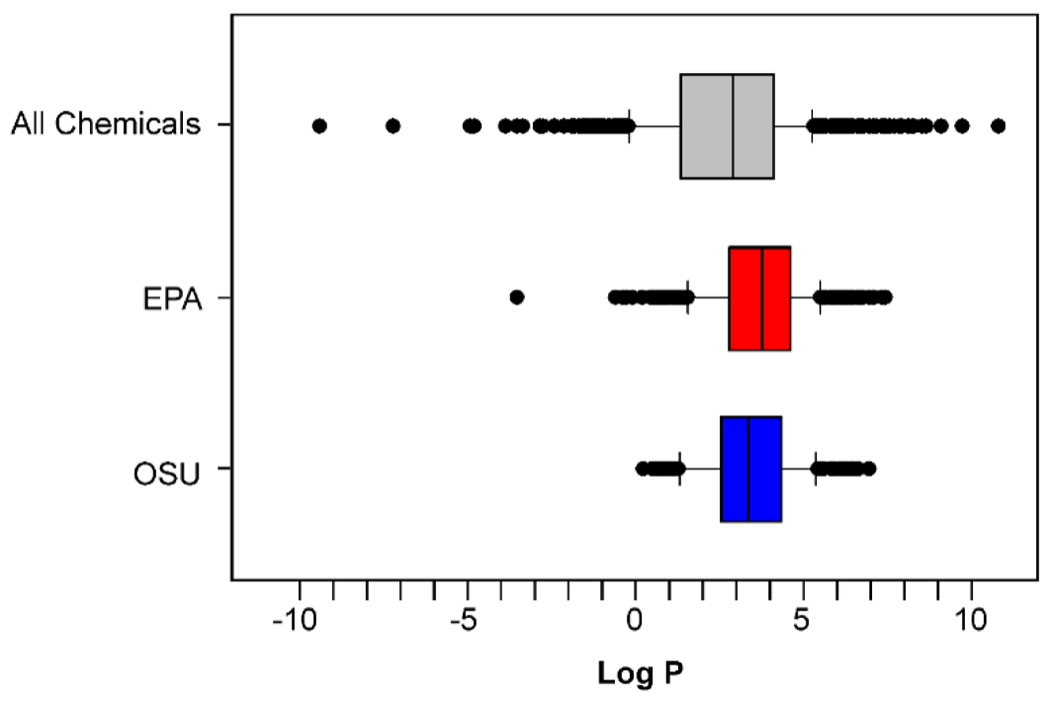
Distribution of active chemicals at EPA and Oregon State University in comparison to logP ToxCast chemicals that were determined to be active at both EPA and Oregon State University (OSU) were plotted based on their logP. The top box plot (grey box) is the logP distribution of all the chemicals in ToxCast Phase I and II. The middle box plot (red box) is the distribution of the actives reported by the EPA laboratory using chorionated embryos and semi-static dosing. The lower box (blue box) is the distribution of the actives reported by OSU using dechorionated embryos and static dosing. Results demonstrate that active chemicals at both laboratories share similar distribution of lipophilicity.

**Fig. 4:**
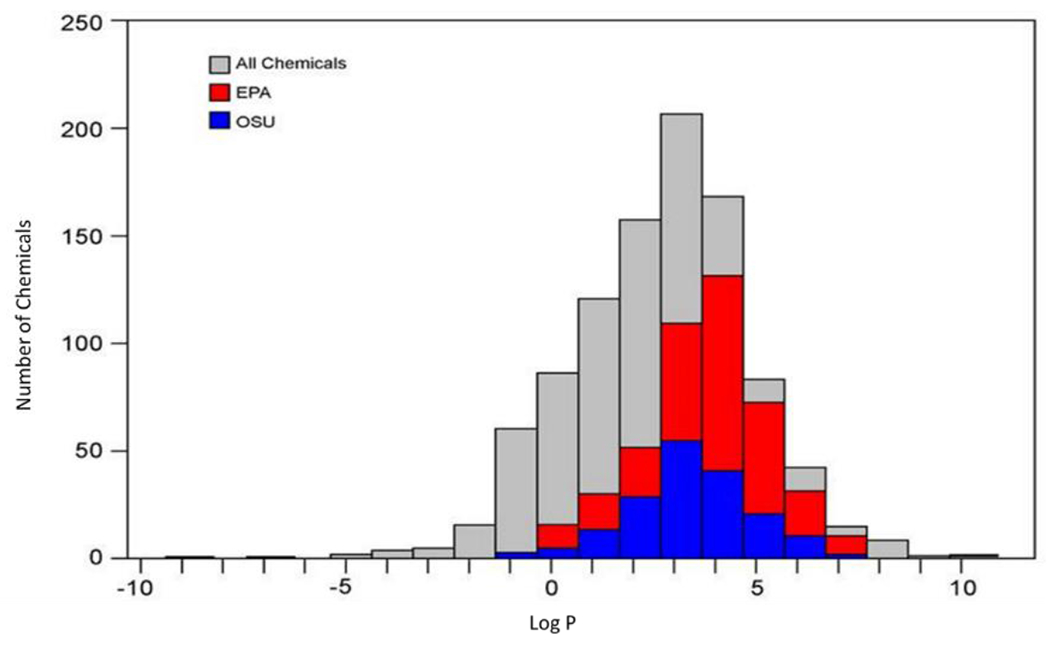
Total numbers of active chemicals as determined by EPA and Oregon State University All overlapping ToxCast chemicals run at both EPA and Oregon State University (OSU) were plotted against logP along with the numbers of active chemicals at each institution. Results demonstrate that a greater number of chemicals were active when using chorionated embryos and semistatic dosing conditions (i.e., EPA protocol).

To date, a comparison of toxicity either within or between laboratories to a defined set of chemicals simultaneously exposed to zebrafish embryos via static and static renewal has not been published. The US EPA and Oregon State University laboratories, however, have tested many overlapping chemicals within the ToxCast effort. While the laboratories differ in a number of experimental design parameters, differences in the status of the chorion and exposure frequency are considered the most relevant to study outcome. Unpublished data from these laboratories suggests that regardless of design parameters, the laboratories identify biologically active chemicals within a similar chemical space (Figure 3). However, repeated dosing appeared to be associated with a greater frequency of active chemicals (Figure 4), a hypothesis that needs to be confirmed with controlled studies given the differences between the two laboratories regarding experimental design variables (unpublished data).

#### 3.3.2 Chorion status

**Fig. 5:**
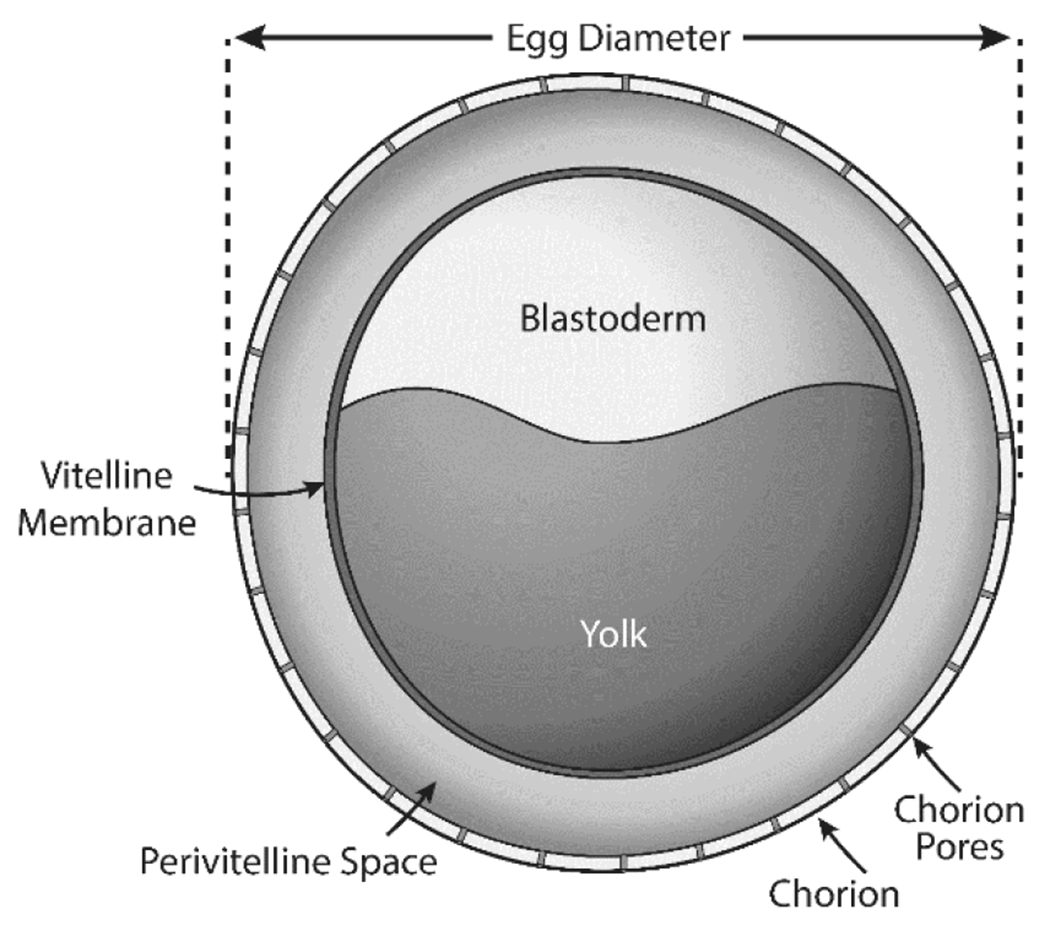
General structure of the zebrafish egg The zebrafish egg is approximately 0.7mm in diameter. The chorion (outer layer) has a thickness of 3.5μM and is fenestrated with 0.5 μM diameter pores allowing passage of water, ions, and chemicals. The fluid-filled perivitelline space overlays the vitelline membrane, which surrounds the yolk and the blastoderm, which will become the developing embryo. Figure adapted from (Jones et al., 1978).

The general structure of zebrafish eggs is shown in Figure 5. The outermost membrane, the chorion, overlays the viscous fluid-filled perivitelline space, the vitelline membrane, and the blastoderm (or embryo) and yolk (Jones et al., 1978; Rawson et al., 2000). The chorion is an acellular envelope (Bonsignorio et al., 1996) about 3.5 μM thick (Rawson et al., 2000). It is highly fenestrated with pores that are approximately 0.5 μM in diameter (Hisaoka, 1958; Rawson et al., 2000), which have been shown to block the movement of molecules in excess of 3 kDa molecular weight (Creton, 2004). In addition to molecule size, the ability of a chemical to pass through the chorion is also affected by its physiochemical properties (Kim and Tanguay, 2014), ionic charge (Cameron and Hunter, 1984; Finn, 2007), electrostatic properties (Burnison et al., 2006), and the concentration of DMSO used in the test system (Kais et al., 2013). For these reasons, some investigators use microinjection techniques to bypass the chorion (Janik et al., 2000) or mechanical, enzymatic, or automated robotic approaches to remove the chorion to ensure the embryos are exposed to test chemicals.

Four of the six IGG investigators use microinjection in their laboratory work, but none use it routinely to deliver test chemical to zebrafish embryos. Members indicated that they found microinjection to be an unacceptable source of uncertainty given that the volumes used are extremely small (nL). Additionally, there is a wide variation in the inner diameters of both handmade and commercially available microinjection needles, making it difficult to deliver consistent volumes of chemical to the embryos. The IGG members also expressed a concern that injected chemicals may not enter the embryo, but instead might partition to the yolk or get trapped in the perivitelline space.

All IGG members indicated that they had tried either mechanical (Henn and Braunbeck, 2011) or enzymatic (Mandrell et al., 2012a) chorion removal. Two laboratories reported that they dechorionate regularly. One laboratory indicated that if a comparison between exposed dechorionated and chorion-intact embryos detected exposure-related differences in their initial studies, dechorionated embryos were used in subsequent assays. One laboratory that uses chorion-intact embryos stated that removal of the chorion very rarely has any effect in their experience. Another laboratory stated that for exposure of nanomaterials, removal of the chorion can be critical.

IGG members from the four laboratories that used chorion-intact embryos routinely expressed a number of concerns:

- Dechorionation adds extra time and cost and reduces throughput.
- Dechorionated embryos are fragile and require either special equipment or training to avoid damaging the embryos when manipulating them.
- Dechorionated embryos stick to the nylon mesh plate inserts used in the IGG member’s laboratory resulting in mortality.
- Dechorionation changes the embryos’ orientation in the well and can interfere with image capture.
- An increase in background malformation and death is observed in dechorionated embryos (Henn and Braunbeck, 2011; Mandrell et al., 2012b).
- Chorion removal prevents the use of hatching as an endpoint.
- Chorion removal is associated with altered behavior in embryonic, larval, and adult fish (Ninness et al., 2006; Thomas et al., 2009).

These investigators pointed out that the literature on the utility of dechorionation is divided, with some laboratories indicating that the chorion might be more permeable than previously believed (Wiegand et al., 2000; Gustafson et al., 2012), and permeability may be affected by solvents like DMSO (Kais et al., 2013).

One laboratory that routinely dechorionates embryos has an automated method for dechorionation and placement of embryos that avoids the technical limitations of traditional approaches to dechorionation (Mandrell et al., 2012a) described above.

The IGG investigators agreed that use of dechorionated embryos is a key factor in experimental design with the potential to affect the outcomes and conclusions of exposure experiments. However, there are few systematic examinations of the utility of chorion removal in the literature. As previously mentioned, Panzica-Kelly (2015) reports slight increases in sensitivity with chorion removal and treatment. Henn and Braunbeck (2011) investigated removal of the chorion to improve the FET test.

Their protocol used a static exposure method to test toxicity of Luviquat HM 552, an aqueous solution of cationic polymers of approximately 400 kDa in size (BASF, 2011), which are too large to readily cross the chorion. The study found that dechorionation increased toxicity of these extremely large polymers. On the other hand, a consortium of biopharmaceutical companies that investigated the uptake of test chemicals found that, while there were chemicals with poor (≤5%) embryo uptake, increasing the maximum test chemical concentration to 1000 μM improved teratogen detection without requiring chorion removal (Ball et al., 2014).

A key finding from the IGG interviews and literature survey was that there are no studies examining the toxicity of a diverse chemical set in chorion-intact and dechorionated zebrafish embryos in which the same chemicals are compared simultaneously in multiple laboratories with accompanying uptake studies.

## 4 Discussion

The 2014 Collaborative Workshop on Aquatic Models and 21st Century Toxicology (Planchart et al., 2016) highlighted the variability that currently exists among participating laboratories with respect to animal husbandry and protocols for chemical screening using zebrafish embryos. Previous efforts to evaluate different zebrafish methods have recommended harmonized protocols (Beekhuijzen et al., 2015). However, the SEAZIT team felt that given the diversity of experimental conditions in use for these studies, forcing researchers to use a single, unified protocol was impractical and that the identification of key factors producing variability in assay outcomes was of the most use to researchers.

All of the IGG laboratories emphasized the importance of using genetically diverse fish stocks, implementing good husbandry practices with high-quality water and feed, maintaining consistent light/dark cycles for adult fish, and avoiding overbreeding of stock fish to obtain quality embryos. The laboratories also emphasized the importance of monitoring breeder fish fecundity, both to ensure availability of sufficient quantities of embryos for testing and as a general indicator of overall fish health.

IGG members described a diverse group of endpoints their laboratories utilized for the comprehensive interrogation of chemical toxicity in zebrafish. Among the IGG laboratories, there is variability in endpoints measured, the means of data capture and scoring, and the interpretation of phenotype alterations. Ducharme et al. (2013) recently surveyed zebrafish developmental toxicity studies and reported a high degree of variability in data collected in these studies. Zhang et al. (2017), using the data from Truong et al. (2014), recently reported an analysis method that develops weighting factors for endpoints, improves prediction of toxic effects, and provides evidence for developmental connections between highly correlated endpoints. Following on these findings, the SEAZIT project is attempting to define best practices for data capture, analysis, and reporting.

The IGG identified two specific parameters that can potentially influence study outcomes for chemical screening using zebrafish embryos: the presence versus absence of the chorion and the use of the static versus static-renewal exposure procedure. IGG members also agreed that, regardless of protocol design, a better understanding of chemical uptake in the zebrafish embryo model would greatly improve the utility of this model.

The input from the IGG members is currently being used to design an interlaboratory study to evaluate the effects of the chorion and exposure methods. Such a study is important to further define the role of protocol design elements in study outcome prior to any attempt to develop one or more harmonized protocols for zebrafish embryos tests. Comparisons of toxicity estimates across laboratories for a diverse chemical set with complimentary data on chemical uptake should allow the further refinement of best practices for a testing protocol(s), adoption of data standards, shared/common endpoints, use of ontologies and data mapping, and standards for data reporting that includes raw data. While the eventual development of a more defined zebrafish embryo test(s) for specified purposes would likely require the development of a well-defined protocol, the current effort focused on defining a set of parameters that all researchers should consider before they start to develop their assay and when publishing results. These advancements would greatly improve the ease with which zebrafish embryo screening data can be used to help inform a broad range of health-related research areas, including chemical hazard assessments. As the SEAZIT effort progresses, we continue to make connections with zebrafish researchers from the U.S. and overseas to collect a broad range of opinions and foster greater consistency in the experimental approaches employed.

## Figures and Tables

**Tab. 1:** Information gathering group members

Member	Affiliation
Stephanie Padilla*	Research Toxicologist Integrated Systems Toxicology Division National Health and Environmental Effects Research Laboratory Office of Research and Development, EPA
Edward Perkins	Senior Scientist Environmental Laboratory U.S. Army Engineer Research and Development Center
Antonio Planchart	Assistant Professor Department of Biological Sciences and Center for Human Health and the Environment North Carolina State University
Donald Stedman	Senior Principal Scientist Pfizer Pharmaceuticals
Tamara Tal*	Biologist Integrated Systems Toxicology Division National Health and Environmental Effects Research Laboratory Office of Research and Development, EPA
Robert Tanguay	Distinguished Professor Department of Environmental & Molecular Toxicology Oregon State University
David Volz	Associate Professor Department of Environmental Sciences University of California, Riverside
Mitch Wilbanks**	Research Biologist Environmental Genomics and Systems Biology Team U.S. Army Engineer Research and Development Center

* Stephanie Padilla and Tamara Tal utilize the same animal facility at EPA and many of their responses were combined to reflect the shared practices.

** Mitch Wilbanks was interviewed and provided responses on laboratory procedures for the U.S. Army Engineer Research and Development Center.

**Tab. 2:** Zebrafish strains used in participating laboratories

Strain Name	Source	IGG Comment
AB	Aquatic BioSystems	
AB	ZIRC	Not very fecund
Modified AB	In-house developed	Recently back-crossed to fish from ZIRC
5D	5D Tropical	Selected for high fecundity
*fli1:egfp* line (reporter gene strain)	Other laboratory	
Tübingen	Not provided	
Outbred wildtype strain	In-house developed	A mixture of several fish obtained from commercial and other laboratory sources (ZIRC)
Wildtype strain	In-house developed	Periodically back-crossed to fish from Seagrest Farms

**Tab. 3:** Adult and larval feeds used at participating laboratories as reported during the IGG interviews

Laboratory	Prepared Diet	Suppliers	Live Food
1^a^	Zeigler Adult ZF Diet Ziegler Larval ZF Diet	Zeigler Bros, Inc. (Gardner, PA)	Artemia (adults)
2 and 3^b^	GEMMA micro	Skretting (Tooele, UT) Reed Mariculture Inc. (Campbell, CA)	Artemia (adults and larvae) Rotifers (larvae)
4	In-house derived, described in (Miller et al., 2014)	NA	None
5	Zeigler Adult ZF Diet	Zeigler Bros, Inc. (Gardner, PA) Reed Mariculture Inc.(Campbell, CA)	Artemia (adults) AP Breed RG Complete Rotifers (larvae)
6^c^	Aquatox Flake	Zeigler Bros, Inc. (Gardner, PA)	Artemia (adults and larvae)
7^d^	Othohime Pellet Zeigler Larval AP 100	Reed Mariculture Incorporated (Campbell, CA) Pentair (Cary, NC)	INVE Artemia (adults and larvae)

a Laboratory 1 now uses GEMMA Micro 75 as a larval diet and GEMMA Micro 300 as an adult diet and has discontinued the use of *Artemia*.

b Laboratories 2 and 3 share the same fish facility and use the same feed.

c Laboratory 6 is phasing out the Aquatox Flake and live food and is switching to GEMMA.

d Laboratory 7 now uses Gemma Micro 75 for larvae up to 20 dpf, Gemma Micro 150 for juveniles up to 60 dpf, and Gemma 300 for adults, and no longer uses live food.

**Tab. 4:** Water parameters in IGG laboratories

Parameter	Range
Water temperature	26.0 to 28.5 °C
pH	7 to 8
Ammonia*	0.001 to 0.8 ppm
Nitrites*	0 to 0.1 ppm
Nitrates*	0 to 20 ppm
Chlorine	undetectable
Salinity	<1 ppm
Dissolved oxygen	>4 ppm
Conductivity	200 to 1248 μS

Abbreviations: C = Celsius; ppm = parts per million; ppt = parts per thousand; μS = microsiemens

* Some laboratories did not measure ammonia, nitrites, or nitrates separately, but reported values as total nitrogen.

**Tab. 5:** Common fish diseases observed at participating laboratories

Disease Name	Causative Orgamsm(s) or Conditions
Noninfectious nephrocalcinosis	High CO_2_ (e.g., > 12 mg/L) in water or excessive levels of calcium and magnesium in the diet. The use of calcium carbonate (rather than sodium bicarbonate) to buffer water in recirculating systems has been associated with the condition.
Gill epithelial hyperplasia	A number of causes including protozoal, parasitic, and bacterial infection; also poor water quality (high ammonia, nitrite, etc).
Egg-associated inflammation and fibroplasia	Unclear, possibly abnormal egg retention and absorption; in some cases, may be due to infection with Mycobacteria.
Mycobacteriosis	*Mycobacteria marinum, M. chelonae, M. fortuitum*, and other species
Piscinoodinium	*Piscinoodinium pillulare*.
Microsporidiosis	*Pseudoloma neurophila*
Aerocystitis pseudoloma	*Pseudoloma neurophilia*

**Tab. 6:** Tissue culture plate size, typical media volume, and number of embryos per well

Plate Size	Volumes Reported	Number of Embryos
T25 and T75 culture flask	25 – 30 mL	10-50
1.5 mL glass plates	250 μl	1
24 well plates	1 – 2.5 mL	1-5
96 well plates	0.25 mL	1
384 well plates*	0.05 mL	1

* The laboratory that uses 384-well plates indicated that development of the embryo limits the length of the exposure period to ~72 hpf. Larva are approximately ~3 mm long and the size of the wells of a 384-well plate is insufficient to house larvae for a prolonged time.

## Footnotes

1 Abbreviations *Dar*T, 48-h embryo test with the zebrafish *Danio rerio;* DMSO, dimethyl sulfoxide; dpf, days post-fertilization; EPA, U.S. Environmental Protection Agency; FET, fish embryo acute toxicity; hpf, hours post-fertilization; IGG, SEAZIT information gathering group; logP, the affinity of a chemical for either aqueous and lipophilic solvents; NICEATM, NTP Interagency Center for the Evaluation of Alternative Toxicological Methods; NTP, National Toxicology Program; OECD, Organisation for Economic Co-operation and Development; SEAZIT, NTP Systematic Evaluation of the Application of Zebrafish in Toxicology; Tu, Tübingen zebrafish strain; ZET, zebrafish embryo teratogenicity

3 doi:10.14573/altex.1804162s
